# A Case of Fahr’s Disease Presenting as Chorea Successfully Treated by the Use of Quetiapine

**DOI:** 10.4137/ccrep.s3423

**Published:** 2009-10-22

**Authors:** Syoichiro Kono, Yasuhiro Manabe, Tomotaka Tanaka, Daiki Fujii, Yasuko Sakai, Hisashi Narai, Nobuhiko Omori, Koji Abe

**Affiliations:** 1 Department of Neurology, National Hospital Organization Okayama Medical Center, Okayama, Japan.; 2 Department of Neurology, Graduate School of Medicine, Dentistry and Pharmaceutical Sciences, Okayama University, Okayama, Japan. Email: ymanabe@okayama3.hosp.go.jp

**Keywords:** Fahr’s disease, basal ganglia calcification, quetiapine

## Abstract

We report a case of 30-year-old man presenting chorea in his legs. A brain computed tomography (CT) scan showed bilateral symmetric calcifications in the basal ganglia, thalamus, cerebellum and subcortical white matter. Laboratory studies showed no abnormalities of serum calcium, phosphate, PTH, lactic acid, pyruvic acid and cerebrospinal fluid. Under the diagnosis of Fahr’s disease (FD), we treated with quetiapine (75 mg/day), which completely abolished his symptoms and he showed no other side effect. Our experience suggests that quetiapine is well tolerated in FD patients and effectively treats chorea without extrapyramidal movement.

## Introduction

Fahr’s disease (FD) refers to a sporadic or familial idiopathic calcification of the basal ganglia that may lead to neurological, psychiatric, and cognitive abnormalities. Patients with FD mostly present with movement disorders such as parkinsonism, chorea, tremor, dystonia, dysarthria, paresis, or speech impairment.^1^,^2^ Other common neurological features are seizures, syncope, or stroke-like events, often combined with a frontal subcortical pattern of behavioral dysfunction and psychiatric symptoms such as psychosis, mood disorders, and dementia.^3^,^4^ The origin and pathophysiology of this disorder are unknown, as is the reason why other cases with basal ganglia calcification remain asymptomatic.^5^,^6^ Treatment of FD is not fully documented. We report a case of FD presenting chorea successfully treated by the use of quetiapine, atypical antipsychotic drug.

## Case Report

**Figure 1:**
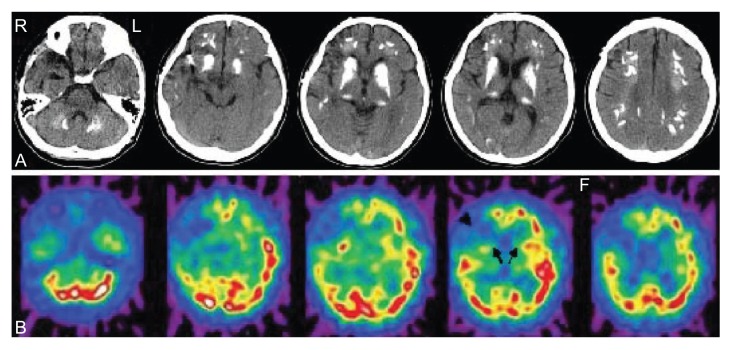
Brain CT showing calcification in the dentate nuclei of the cerebellum, basal ganglia and frontal white matter, and the central semiovale **A**. SPECT using ^123^I-ECD scan (transverse slices) showing hypoperfusion in the basal ganglia (arrows) and right frontal lobe (arrowhead) **B**.

A 30-year-old man was admitted with choreiform movements of his legs which disappeared during sleep. He presented generalized tonic-clonic seizures at age 14. He was treated with sodium valproate at 600 mg/day. Brain CT showed calcification in the basal ganglia and frontal white matter at age 15. The symptoms did not progressed. He was treated with removal of right temporal lobe hematoma and right middle cerebral artery aneurysmal neck clipping at age 28. He presented auditory hallucinations and paranoid delusion at age 29. Four mg of risperidon a day stabilized his psychotic symptoms. After 2 months of risperidon maintenance the auditory hallucinations and paranoid delusion disappeared. However, he complained of an extrapyramidal gait disorder. His risperidon dosage was decreased gradually for one month and stopped one month before admission. None of his family suffered similar symptoms or had histories of dementia, movement disorders or other neurological and psychiatric diseases. On admission, the patient did not retard development. On neurological examination, he was alert and oriented without any cognitive deficits. Choreiform movement on his both (left side dominant) lower limbs was observed. There were no obvious motor or sensory abnormalities, and his reflexes were equal bilaterally in both the upper and lower limbs. Albright’s sign (short stature, round face, and short metacarpal and metatarsal bones) was negative. Physical examination was unremarkable. Laboratory and endocrinological investigations were all normal. In particular, screening for hypoparathyroidism, iron or copper deficiencies, mitochondrial encephalopathies, encephalitis, and Creutzfeld-Jakob disease were all negative, and serum and urine calcium metabolism was normal. No thyroid disease or vitamin deficiency could be found. Serologic tests for syphilis and HIV were negative. Cerebrospinal fluid study was normal. The electroencephalography (EEG) was normal. Brain CT and MRI after admission documented marked and symmetrical calcification in the dentate nuclei of the cerebellum, basal ganglia, frontal white matter, and the central semiovale (Fig. 1A). Single photon emission computed tomography (SPECT) using ^123^I-ECD (transverse slices) showed hypoperfusion in the basal ganglia and right frontal lobe (Fig. 1B). The patient began to be treated with tiapride (150 mg/day for one week). However, choreiform movement on his both lower limbs persisted. We stopped to give tiapride and fifty mg of quetiapine a day partially stabilized the chorea and showed no other side effects. With a dose of 75 mg/day, his chorea completely disappeared and have no recurred. We continued quetiapine treatment.

## Discussion

FD is a rare clinical entity characterized by non-arteriosclerotic calcification of the striopallidodentate system bilaterally without blood calcium or hormone level abnormalities.^1^ Although treatment of underlying etiologies (such as hypoparathyroidim or mitochondrial encephalopathy) has led to neuropsychiatric improvement, there are no specific treatments to limit calcification progression to our knowledge.^2^ There are a number of case reports about the usage of risperidone, quetiapine, olanzapine and also haloperidol, both for chorea and psychotic symptoms in Huntington’s disease.^7^ Several groups have demonstrated that quetiapine may be effective to treat drug-induced dyskinesias in Parkinson’s disease.^8^,^9^ However, the treatment for chore in FD is not fully documented.

In our patient, quetiapine (a dopamine receptor blocking agent) successfully ameliorated chorea. There is little possibility of the withdrawal of risperidone, because the patient persisted chorea for one month after stopping risperidone. The choeriform movement was left sided dominant. Although SPECT showed right sided dominant hypoperfusion owing to right temporal lobe hemorrhage, this left sided dominant choreiform movement may be related with right sided dominant hypoperfusion in SPECT. Although calcifications can involve other structures as well, the globus pallidus is most commonly involved.^1^ Defective iron transport and free radical production may damage tissue, initiating calcification. The mineral and biochemical content as well as the histopathological correlates of calcifications have been defined. Mineral composition varies by anatomic site and proximity to vasculature. The mechanism by which quetiapine may have beneficial effects in FD is unclear. Preferential loss of D2 projection neurons, which are involved in a feedback loop normally active in the suppression of involuntary movements, is thought to be the pathophysiologic basis of FD-chorea. The D2 antagonist properties of quetiapine may explain its benefits. However, the effects at other actions of quetiapine such as adrenergic and serotonergic systems may also be useful. Our experience suggests that quetiapine is well tolerated in FD patients and effectively treats chorea without extrapyramidal movement. Controlled, long-term studies of this and other atypical neuroleptics in the treatment of movement disorders in patients with FD are warranted.
